# Read, use and cite

**DOI:** 10.1093/jhps/hnaa032

**Published:** 2020-09-29

First the good news. We’ve done it. Together, and it has been a huge team effort, we have reached the point when *JHPS* has been awarded its first impact factor (IF). It may be 1.917, when there are so many with IFs higher than this, but there are even more with IFs that are lower, and masses with no IF at all. This first IF puts us in the top 50% of orthopaedic journals, which is truly amazing. Thank you to everyone who has been involved in this Herculean task. For a first IF, this value is excellent. It is now up to us all to keep on climbing. There is no limit to what our IF could be as long as each of us uses *JHPS* as much as we can and we cite, cite, cite.

Next the bad news, or at least my worry, brought about by COVID-19. Publishing is undergoing a real transformation. The UK journalism industry faces a loss of more than £1 billion in 2020. Broken down, advertising revenue is forecast to fall some £570 million for newspapers, £200 million for magazines and £210 million for digital [1]. There is no let-up in sight [2]. Dennis Publishing, which owns many magazines covering automotive, current affairs, lifestyle and technology, has recently placed a quarter of its UK staff into a redundancy consultation process [3]. Bauer Media, which is German-owned, is another large publisher that is thinking of doing the same [4]. This publisher reaches more than 22 million adults in the UK. I fear that news like this is just the start and just one small part of one country. The USA is no different. Barnes and Noble closed 500 stores and furloughed thousands of employees, MacMillan has laid off staff and cut employees’ salaries, hundreds of independent bookshops are struggling to survive, while Amazon has deprioritized its warehouse space for books and pushed back delivery dates for many print titles [5]. In India, mainstream publishing is undergoing its worst crisis in living memory [6]. I wager I can say the same about any country I might choose.

Academic publishing is no different, as publishing houses are also forced to rationalize what they do. Furthermore, and I am certain many have noticed, the pandemic appears to be the era of the pre-print server. MedRχiv [7] (pronounced ‘med-archive’) is a popular choice. Pre-print servers are locations where research can be published without peer review and simply stay there to be read by the world. The work can subsequently be submitted to a peer-reviewed journal and, even if the submission is rejected, the research can remain on the pre-print server. Many of the so-called important research developments during the COVID-19 pandemic have come from research that has not been peer-reviewed. However, what these pre-print servers allow is speedy publication at a time when information needs to be widely disseminated. They work at a pace with which peer-reviewed journals simply cannot compete. The downside is the lack of a peer-review safeguard. Yet the pandemic has shown just how fast and open science publishing can be—when scientists want it that way [8]. I suspect we will look back on these times and say that COVID-19 was when academic publishing changed. I, for one, will look at the use of pre-print servers very differently now, compared with how I perceived them barely 6 months ago.

Some findings have not even made it as far as a pre-print server. Not so long ago I was hearing that dexamethasone was the way forward for COVID-19 infection. I heard this at a government news briefing and the finding was declared a major breakthrough [9]. Dexamethasone was said to reduce in-hospital deaths by a third in patients with severe respiratory complications, or so the RECOVERY Trial [10] from Oxford reported. Yet I learned that from a politician without a medical qualification to his name. I did not read about it in a peer-reviewed journal.

Thanks to the rush to be published during the pandemic, journals seem to be falling over each other to publish papers on COVID-19. Because of this, oddities can slip through, even for well-established journals. Retraction Watch [11] is one of my favourite web sites, which I must visit at least five times weekly. As I write, the site reports 22 papers on COVID-19 that have been retracted, three have been temporarily retracted, and there are two expressions of concern. Some are papers on preprint servers, others are more traditional. For example, there was the hydroxychloroquine study that appeared in *The Lancet* on 22 May 2020 but had been retracted by 4 June 2020 [12]. For a period, this article was the death knell of hydroxychloroquine in the management of COVID-19 and resulted in the cessation of a World Health Organization (WHO) study into the drug. *The Lancet* has an IF of approximately 59.

Or, the letter by Bae *et al*. [13] from South Korea in the *Annals of Internal Medicine*. The letter declared that both surgical and cotton masks were ineffective in preventing dissemination of severe acute respiratory syndrome coronavirus 2 (SARS-CoV-2) from the coughs of patients with COVID-19, to the environment and external mask surface. The letter first appeared in the journal on 6 April 2020 and had been retracted by 1 June 2020. The journal’s IF is approximately 20. At the time of writing this editorial, the letter has had what is called an Attention Score of 7213. This puts it in the top 5% of all research scored by Altmetric. To date, the letter has been mentioned by 104 news outlets, 15 blogs, two policy sources, 10 498 Tweeters and 34 Facebook pages. The demographic breakdown of the Tweeters showed that 89% of them were members of the public but 10% were scientists or healthcare practitioners of some sort [14].

It thus appears the pandemic has changed the face of scientific research. Faulty papers are slipping through the net, perhaps because of the haste to publish. Pre-print servers, which are not peer-reviewed, are dictating policy and may sometimes be the first port of call for those who wish to be updated on current research. Announcements are made by politicians about scientific research that has not even been submitted. Where will it all end? What seems clear is that the world of academic publishing once COVID-19 comes under control, if such a Utopia is ever possible, will be very different to the world we experienced beforehand. It is why publishers are proceeding with great caution, and why no journal can rest on its laurels and presume that in a year’s time it will still exist.

What about *JHPS*? So far so good. Our first IF is brilliant, and it is thanks to everyone that we have got this far. My job, your job, all our jobs, is to secure our future as best we can. For this reason, I would beg of you all to read us, use us and cite us, definitely cite us, wherever and whenever you can. I should add that I am delighted to say that our submissions are increasing at a time when I thought they might decline. Please keep it that way.

Turning to our last issue, number 7.1, it has proved welcome reading for me during the pandemic to date. I was particularly fascinated by the paper from McGovern *et al*. [15] that supported the use of conservative management to improve outcomes for patients with pre-arthritic hip pain. Also, and because I am an enthusiast for the use of fibrin, I always like to read of those who handle articular cartilage defects in different ways. The paper by Arriaza *et al*. [16] did this. The senior author had, first time round, elevated an articular cartilage defect that presented as a wave sign, undertaken a subchondral microfracture and fixed the adjacent labrum with bone anchors. This second-look study showed that although 85% had a wave sign on the first occasion, only 15% had it on second look. I am not sure I will put my fibrin away at the moment but may have to start looking at it with suspicion. We will see.

As for this issue, number 7.2, once again it is impossible for me to choose. Basically, each paper has held me spellbound, but then you would expect that of an editor. Completely subjectively, and without apology, two stand out to me. First, and especially as the number of submissions we are receiving in the field of open hip preservation surgery is slowly rising, I saw the paper by Lara *et al*. [17] on Bernese periacetabular osteotomy. They looked at patients who had undergone an osteotomy but whose intra-articular lesions had remained untreated. They obtained excellent results from a periacetabular osteotomy but without labral repair. That flies in the face of what I was expecting but *JHPS* is all about the unexpected.

The other paper that had me nodding in agreement was that by Waryasz *et al*. [18] who looked at whether patients understand what we are doing to them. I would like to pretend that I did not expect their findings. The authors concluded, and I am citing from their paper, ‘Although we made significant pre-operative oral and written efforts to help patients achieve an elementary level of health literacy regarding their forthcoming hip arthroscopy, many patients did not achieve satisfactory comprehension’. Why does that not surprise me?

So, as ever, please enjoy this issue of *JHPS*. It is published for you, the hip preservation practitioner, and is filled from cover to cover with brilliance. I commend this issue to you in its entirety.

Oh yes, and please read, use and cite this journal at every opportunity. Ask everyone you know to do the same.

My very best wishes to you all.

**Richard (Ricky) Villar**

*Editor-in-Chief, Journal of Hip Preservation Surgery*
